# Clinical Validation of a New Optical Biometer for Myopia Control in a Healthy Pediatric Population

**DOI:** 10.3390/children9111713

**Published:** 2022-11-09

**Authors:** Elena Martínez-Plaza, Ainhoa Molina-Martín, Alfonso Arias-Puente, David P. Piñero

**Affiliations:** 1 Group of Optics and Visual Perception, Department of Optics, Pharmacology and Anatomy, University of Alicante, 03690 Alicante, Spain; 2 University of Valladolid, 47001 Valladolid, Spain; 3 Department of Ophthalmology, Vithas Medimar International Hospital, 03016 Alicante, Spain

**Keywords:** axial length, biometry, Myah device, myopia control

## Abstract

To assess the clinical validation of the Myah device in a pediatric population by evaluating the repeatability of biometric evaluations and analyzing its agreement with the Myopia Master system. A total of 51 children (51 eyes) were enrolled. Repeated measurements of flat (K1) and steep (K2) corneal radius, white-to-white (WTW) distance and axial length (AL) were performed with the Myah device. The same parameters were obtained from a subgroup (30 eyes) with the Myopia Master for the agreement analysis. The repeatability was assessed using the intrasubject standard deviation (Sw) and the intraclass correlation coefficient (ICC). The agreement was analyzed using the Bland–Altman method and the paired Student *t*-test. The Sw was 0.018 D, 0.021 D, 0.071 mm and 0.017 mm for K1, K2, WTW and AL, respectively (ICC ≥ 0.971). The mean difference and limits of agreement when comparing instruments were −0.013 (−0.102/0.077) for K1 (*p* = 0.16), −0.058 (−0.127/0.012) for K2 (*p* < 0.001), 0.151 (−0.370/0.673) for WTW (*p* < 0.001) and 0.030 (−0.091/0.151) for AL (*p* = 0.009). In conclusion, the Myah device provides consistent measurements of corneal radius, WTW distance and AL in a healthy pediatric population, validating their usefulness in clinical practice. These measurements could be used interchangeably with those provided by the Myopia Master device, although with some caution.

## 1. Introduction

The increasing prevalence of myopia has been deeply described in recent years, estimating that half of the global population will be myope in less than 30 years, with 10% even above −5 diopters (D) [1]. Environmental factors, including near work or low outdoor exposure, have been related to myopia onset and progression [1]. Moreover, the recent advancement in genetic analysis has allowed the identification of several genes involved with myopia predisposition, demonstrating its hereditary component [2]. In addition, it is widely known that there is a close relationship between high myopia and suffering irreversible posterior segment pathologies, such as myopic maculopathy or retinal detachment, among others [3,4,5]. In an attempt to avoid or reduce myopia progression and, consequently, the associated pathologies, several strategies for myopia control in children have been developed. Indeed, reducing 1 D of myopia is estimated to reduce about 40% the probability of suffering from myopic maculopathy later in life [6], highlighting the relevance of myopia control.

Myopia progression is mainly related to an elongation of the anterior–posterior axial dimensions of the ocular globe. For that reason, the strategies for myopia control focus their efficacy on reducing the progression of the refractive error by controlling the axial length (AL) [7]. In fact, the FDA Workshop for “Controlling the Progression of Myopia: Contact Lenses and Future Medical Devices”, that was held in 2018, suggested that the primary effectiveness endpoint for future studies and clinical trials should be, preferably, the AL [8]. Hence, the use of ocular biometric devices is indispensable in the study and control of myopia.

Given the growing interest in myopia control, modern biometric devices have been developed and specially focused for use in young myopic patients. The multidiagnostic platform Myah^®^ (Topcon Healthcare Inc., Tokyo, Japan) has been recently launched on the market, allowing one to perform optic biometry. Particularly, the Myah device allows the measurement of flat (K1) and steep (K2) corneal radius, white-to-white (WTW) distance and AL. Considering the relevance of measuring the AL correctly for adequate control of myopia progression, clinical validation of these type of devices is crucial from a clinical viewpoint. The Myah device estimates the AL using interferometry technology, which is considered the gold standard [9]. Another device developed for evaluating myopia progression is the Myopia Master^®^ (Oculus GmBH, Wetzlar, Germany), whose repeatability in terms of biometric parameters and its agreement with the IOL Master 500 and IOL Master 700 have been reported in a pre-printed study [10]. The aim of the present study was to assess the clinical validation of the Myah device for the biometric parameters (K1, K2, WTW and AL) in a pediatric population, first by evaluating the repeatability of these parameters, and second, by analyzing the agreement with the available optical biometer, Myopia Master.

## 2. Materials and Methods

The present work was an observational single-visit study performed at the Department of Ophthalmology of Vithas Medimar International Hospital (Alicante, Spain). The study was conducted in accordance with the Declaration of Helsinki and approved by the Ethics Committee of the University of Alicante.

Inclusion criteria were myopia patients with an age equal or less than 18 years and a healthy eye according to the complete eye examination performed. Exclusion criteria were previous ocular surgery, presence of any ocular pathology or systemic disease, or the use of topical medications.

### 2.1. Clinical Measurements

A complete eye evaluation involving manifest refraction, corrected distance visual acuity (CDVA) and slit-lamp evaluation for both eyes was performed by the same experienced clinician (D.P.P.). After this examination, optic biometry was performed in all subjects using the Myah device. In addition, a subgroup of subjects was also evaluated with the Myopia Master device. One eye per patient was randomly selected for study purposes.

### 2.2. Myah Device

**Figure 1:**
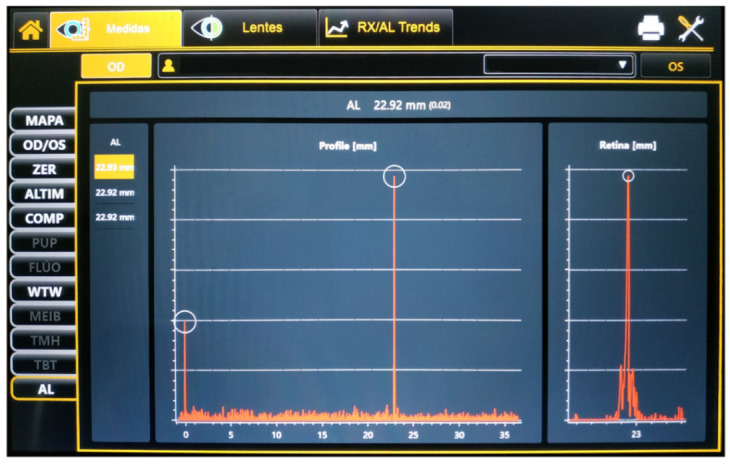
Display of the Myah device after an axial length measurement.

The Myah device is a multidiagnostic platform that includes an optical biometer, a corneal topographer, a pupilometer and a dry eye module. Optic biometric exams were performed, obtaining data from the corneal radius, the flat (K1) and the steep (K2) keratometry, WTW and AL distances (Figure 1). Two consecutive measurements were performed to collect the corneal radius and WTW data. These parameters were obtained using the 24 Placido rings reflected from the anterior corneal curvature. In addition, three consecutive measurements were performed for AL repeatability purposes. The device performs six individual interferometric measurements per exam using low-coherence interferometry (820 nm diode laser) and shows the mean values per each measurement.

### 2.3. Myopia Master Device

**Figure 2:**
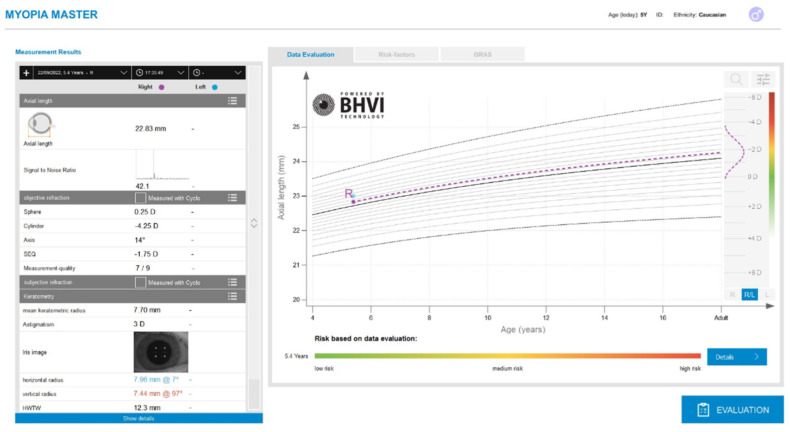
Display of the Myopia Master device after an optic biometric measurement.

The Myopia Master is a biometric device, specifically developed to manage myopia (Instruction Manual Myopia Master^®^ [G/68100/EN Rev04 0820]). A single acquisition was performed, obtaining the corneal radius (K1 and K2), WTW and AL data (Figure 2). The device performs the corneal radius measurement using the integrated keratometer (analysis of the reflections of several projected spots and a ring) and the AL data is obtained using the integrated interferometry technology (the device performs six individual interferometric analyses per exam).

### 2.4. Statistical Analysis

The statistical analysis was performed with the SPSS statistical software version 28.0.0 for Windows (IBM SPSS Inc., Chicago, IL, USA). Normality of data distributions was checked with the Shapiro–Wilk test.

The analysis of variance (ANOVA) for repeated measurements was used to compare the three AL measurements of the Myah device, while a paired Student *t*-test was used to compare the two measurements of K1, K2 and WTW. The intrasubject standard deviation (Sw), precision, repeatability, coefficient of variation (CoV) and the intraclass correlation coefficient (ICC) were calculated for the AL measurements. The Sw was estimated as explained by Bland and Altman [11]. The precision, calculated as 1.96 × Sw, can be interpreted as the difference between a patient’s measurement and the true value for 95% of observations. The repeatability, calculated as 2.77 × Sw, can be interpreted as the difference between two observed measurements with a probability of 95%. The CoV, calculated as the percentage of the ratio of the Sw and the overall mean, can be interpreted as the percentage of dispersion around the mean. The ICC value estimates the reliability, with values below 0.5 indicating poor reliability while values near to 1 indicate excellent reliability. The agreement assessment between measurements was analyzed by using the paired Student *t*-test and the Bland–Altman method. The 95% LoAs were determined as the mean difference of ±1.96 SD. Two-sided *p*-values equal to or less than 0.05 were considered statistically significant.

## 3. Results

A total of 51 eyes (27 right eyes and 24 left eyes) of 51 patients (26 females and 25 males) with a mean age of 10.80 ± 3.40 years (range 5 to 18 years) were including for analyzing the repeatability of the Myah device. The mean spherical equivalent was −1.20 ± 2.47 D (range −6.25 to +5.13 D), and the mean corrected distance visual acuity was −0.01 ± 0.04 logMAR (range −0.10 to +0.10 logMAR).

A subgroup of 30 eyes (15 right eyes and 15 left eyes) of 30 patients (15 females and 15 males) with a mean age of 9.93 ± 2.97 (range 5 to 18 years) was also evaluated with the Myopia Master device, whose data were used to analyze the agreement between the Myah and Myopia Master devices. The mean spherical equivalent of this subgroup was –1.26 ± 1.94 D (range −5.50 to +1.38 D), and the mean corrected distance visual acuity was −0.01 ± 0.05 logMAR (range −0.10 to +0.10 logMAR).

### 3.1. Repeatability of Myah Device

**Figure 3:**
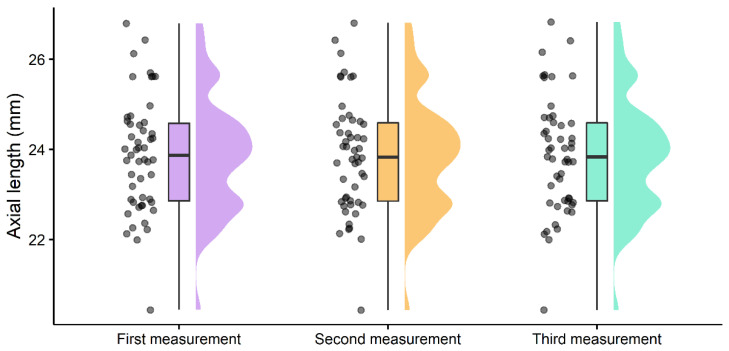
Distribution of the three repeated measurements of axial length performed with Myah device. Each point represents a single measurement. The box and whiskers represent the minimum, first quartile, median, third quartile and maximum values. The density illustration represents the smooth distribution of the data.

The mean values and comparison of each measurement are shown in Table 1. The distribution of the repeated measurements for the axial length is shown in Figure 3. Table 2 presents the Sw, the precision, the repeatability, the CoV and the ICC for the K1, K2, WTW and AL measurements.

### 3.2. Agreement between Myah and Myopia Master Devices

**Figure 4:**
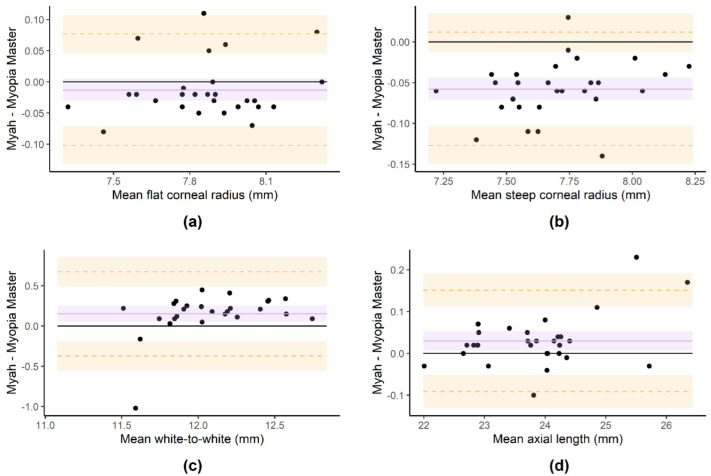
Bland–Altman plots of the flat corneal radius (**a**), steep corneal radius (**b**), white-to-white (**c**) and axial length (**d**) measurements performed with Myah and Myopia Master devices. The purple line and shadow represent the mean difference and its 95% confidence interval, respectively. The orange dashed lines and shadows represent the lower and upper limits of agreement and their 95% confidence intervals, respectively.

The mean values and standard deviation were 7.85 ± 0.23 mm and 7.87 ± 0.23 mm for the K1 measurements; 7.67 ± 0.23 mm and 7.73 ± 0.23 mm for the K2 measurements; 12.19 ± 0.40 mm and 11.99 ± 0.30 mm for the WTW measurements; and 23.86 ± 0.97 mm and 23.83 ± 0.94 mm for the AL measurements using the Myah and Myopia Master devices, respectively. No significant differences were found for the K1 measurement between both devices (*p* = 0.16). Contrarily, the mean value of K2 was significantly higher for the Myopia Master device (*p* < 0.001), and the mean values of WTW and AL were significantly higher for the Myah device (*p* < 0.001 and *p* = 0.009, respectively). Table 3 and Figure 4 show the Bland–Altman data and plots, respectively.

## 4. Discussion

An adequate AL measurement in myopia control patients is crucial for monitoring myopia progression, especially in a pediatric population. For that purpose, new devices have been launched on the market to accurately measure biometric parameters, such as the Myah device, whose clinical validation may be of great interest for eye care practitioners involved in myopia control. To perform such clinical validation, the purpose of the present study was, first, to evaluate the repeatability of the parameters K1, K2, WTW and AL and, second, to analyze the agreement of these biometric parameters with the available optical biometer Myopia Master.

The repeated measurements of K1, K2 and WTW performed with the Myah device showed an excellent ICC, equal to or higher than 0.97 in all cases, obtaining an Sw of 0.02 mm, 0.02 mm and 0.07 mm, respectively. These values of Sw found for the keratometry are similar to those reported using the Lenstar LS900 (Haag-Streit AG, Koeniz, Switzerland) biometer in children, around 0.02 mm for both main corneal meridians [12]. Likewise, the Sw values for the corneal radius reported for the AL-Scan (Nidek Co., Ltd., Gamagori, Japan) and the IOL Master 700 (Carl Zeiss Meditec AG, Jena, Germany) devices were between 0.08 D and 0.11 D in children [13,14]. In the present study, the Sw for the keratometry (K1 and K2), calculated in terms of refractive power, would be of 0.10 D, approximately, a value comparable to that reported using the IOL Master 700 device. Regarding the WTW, the Sw of the IOL Master 700 device has been reported as 0.09 mm [14], a value slightly worse than the one found for the Myah device. Therefore, these results indicate that the repeatability values of the Myah device for the biometric parameters associated with the anterior segment are comparable to those of other biometers. Consequently, the Myah device may be useful for analyzing the corneal geometry in children who need to wear contact lenses, e.g., contact lens fit for myopia control.

Regarding the AL measurements, the repeatability of the Myah device was even better than that of the previous parameters, showing a Sw of 0.02 mm and an ICC of 1. Similarly, studies analyzing the repeatability of the previous biometer available from the same manufacturer, the optical low-coherence biometer named Aladdin (Topcon, Tokyo, Japan), also found consistent measurements of AL in adults [15,16]. In pediatric populations, the Sw for the AL measurement has been reported as 0.03 mm, 0.03 mm and 0.01 mm using the Lenstar LS900, AL-Scan and the IOL Master 700 devices, respectively [12,13,14]. Therefore, the repeatability found for measuring AL with the Myah device in children is comparable with that of other biometers available in the market. The implication of such excellent repeatability is that eye care practitioners involved in myopia control can be confident in using the Myah device to evaluate the progression of myopia in children. In addition, the AL measurement, in combination with the corneal biometric parameters, may also be useful for conditions requiring crystalline lens extraction with an intraocular lens implantation, e.g., congenital cataract [17,18].

The use of the optic biometer Myopia Master in the present study allowed us to analyze the agreement between both instruments (Myah vs. Myopia Master devices). Differences between devices were small in magnitude, but achieved statistical significance for K2, WTW and AL. However, the clinical relevance of such differences is questionable. Specifically, both measurements, K1 and K2, tended to be higher when using the Myopia Master device. Corneal radius and WTW distance are commonly used to select the initial parameters for contact lens fitting (base curve and diameter), whose use is increasing in children due to the recent development of myopia control techniques [19]. The mean difference found for K2 in the present study, 0.058 mm, is around one step for base curve selection in rigid contact lenses. Thus, the difference found between instruments may have some influence on the parameter selection decision when fitting rigid contact lenses. On the other hand, this difference can be considered as negligible for soft contact lens fitting, as the base curve steps are typically higher. Regarding the WTW measurement, the higher values were provided by the Myah device; however, the mean difference between instruments, 0.15 mm, appears not to be of a magnitude high enough to alter the diameter decision for contact lens fitting.

The mean difference for AL measurements between both devices was 0.03 mm, which corresponds with a refractive power difference of 0.1 D approximately. As a result, the present study suggested that the Myah and Myopia Master devices show slightly different values for AL. Therefore, given the importance of an accurate AL measurement for control of myopia progression [7,8], both instruments could be used as interchangeable for that purpose, but with some caution. Nonetheless, considering that, in clinical practice, the lower difference of refractive power that is possible to compensate with ophthalmic lenses is 0.25 D, the difference found in AL between devices would be negligible from a refractive viewpoint.

Other studies analyzing the agreement between optical biometers, in adult cohorts, have also found significant differences in several parameters [20,21,22]. Comparing the Myah device with other biometers, only one study has been developed in a pediatric population [23]. These authors found no significant differences between the Pentacam AXL (Oculus Optikgerate GmbH, Wetzlar, Germany), IOLMaster 700, and Myah devices in terms of AL. In addition, they found differences between these devices in the corneal radius, although these were considered clinically insignificant. The different methodologies that should be applied when using each instrument may be a possible factor interfering in the biometric measurements, e.g., the time spent by each device for performing a measurement. In addition, the use of different technologies may also influence the results obtained, e.g., the corneal curvature can be measured using different technologies, such as the Placido disk or the Scheimpflug camera.

Some methodological aspects of the present study may be considered as a limitation. First, while two devices were selected to accomplish the study’s purposes, several biometers are currently available in the market. The Myopia Master device was selected to compare with the Myah instrument based on the fact that both devices were specifically developed to assess the axial growth, which is particularly of interest in pediatric populations (Topcon Myah^®^–User manual (rev. 3 ES–2021); Instruction Manual Myopia Master^®^ (G/68100/EN Rev04 0820)). In addition, both instruments are included in the Devices@FDA database (https://www.accessdata.fda.gov/scripts/cdrh/devicesatfda/index.cfm accessed on 4 November 2022). However, future studies assessing the agreement between the Myah device and other optical biometers could be of great interest for clinicians. Second, the clinical validation was performed in a pediatric population given the increasing interest of myopia control, although the validation of the Myah device in an adult population would complement the knowledge about the instrument’s performance. Finally, the lack of cycloplegia for biometry measurements could be considered as a limitation. However, it has been already reported that cycloplegia has no influence on axial length or corneal curvature measurements taken with different biometers, both in children and adult populations [24,25].

## 5. Conclusions

The Myah device provides consistent measurements of corneal radius, WTW distance and AL in a healthy pediatric population. Therefore, this device is a useful instrument to monitor myopia progression as well as to adapt contact lenses, e.g., myopia control contact lenses, in children. The Myah and Myopia Master devices showed excellent agreement for the K1 and K2 measurements, whereas the agreement with the other two parameters could be considered as acceptable for clinical purposes. Consequently, the measurements of corneal radius, WTW distance and AL provided by the Myah device in a healthy pediatric population could be used interchangeably with those provided by the Myopia Master device, but with some caution.

## Figures and Tables

**Table 1:** Mean values and standard deviation of the repeated measurements performed with the Myah device.

Parameter	1st Measurement (Mean ± SD)	2nd Measurement (Mean ± SD)	3rd Measurement (Mean ± SD)	*p*-Value
K1 (mm)	7.90 ± 0.27	7.90 ± 0.27	-	0.29
K2 (mm)	7.71 ± 0.27	7.71 ± 0.27	-	0.85
WTW (mm)	12.13 ± 0.41	12.12 ± 0.42	-	0.58
AL (mm)	23.87 ± 1.27	23.87 ± 1.27	23.87 ± 1.27	0.89

AL: axial length; K1: flat corneal radius; K2: steep corneal radius; SD: standard deviation; WTW: white-to-white.

**Table 2:** Intrasubject repeatability for axial length measurement obtained with the Myah device.

Parameter	Sw (95% CI)	Precision (95% CI)	Repeatability (95% CI)	CoV % (95% CI)	ICC
K1 (mm)	0.02 (0.01/0.02)	0.04 (0.03/0.04)	0.10 (0.07/0.12)	0.22 (0.16/0.29)	1.00 (0.99/1.00)
K2 (mm)	0.02 (0.02/0.03)	0.04 (0.03/0.05)	0.06 (0.04/0.08)	0.28 (0.20/0.35)	0.99 (0.99/1.00)
WTW (mm)	0.07 (0.05/0.09)	0.14 (0.10/0.18)	0.20 (0.14/0.25)	0.59 (0.43/0.75)	0.97 (0.95/0.98)
AL (mm)	0.02 (0.01/0.02)	0.03 (0.02/0.04)	0.05 (0.03/0.06)	0.07 (0.05/0.09)	1.00 (1.00/1.00)

AL: axial length; CI: confidence interval; CoV: coefficient of variation; D: diopters; ICC: intraclass correlation coefficient; K1: flat corneal radius; K2: steep corneal radius; Sw: intrasubject standard deviation; WTW: white-to-white.

**Table 3:** Agreement between Myah and Myopia Master devices for K1, K2, WTW and AL measurements.

Parameter	Mean Difference (95% CI)	Lower LoA (95% CI)	Upper LoA (95% CI)
K1 (mm)	−0.01 (−0.03/0.01)	−0.10 (−0.13/−0.07)	0.08 (0.05/0.11)
K2 (mm)	−0.06 (−0.07/−0.04)	−0.13 (−0.15/−0.10)	0.01 (−0.01/0.04)
WTW (mm)	0.15 (0.05/0.26)	−0.37 (−0.55/−0.19)	0.67 (0.49/0.86)
AL (mm)	0.03 (0.01/0.05)	−0.09 (−0.13/−0.05)	0.15 (0.11/0.19)

AL: axial length; CI: confidence interval; K1: flat corneal radius; K2: steep corneal radius; LoA: limit of agreement; SD: standard deviation; WTW: white-to-white.

## Data Availability

Data are contained within the article.
